# Cationic oligonucleotide derivatives and conjugates: A favorable approach for enhanced DNA and RNA targeting oligonucleotides

**DOI:** 10.3762/bjoc.17.125

**Published:** 2021-07-29

**Authors:** Mathias B Danielsen, Jesper Wengel

**Affiliations:** 1 Biomolecular Nanoscale Engineering Center, Department of Physics, Chemistry and Pharmacy, University of Southern Denmark, Campusvej 55, 5230 Odense M, Denmark

**Keywords:** antisense oligonucleotides, backbone modifications, cations, nucleobase modifications, sugar modifications

## Abstract

Antisense oligonucleotides (ASOs) have the ability of binding to endogenous nucleic acid targets, thereby inhibiting the gene expression. Although ASOs have great potential in the treatment of many diseases, the search for favorable toxicity profiles and distribution has been challenging and consequently impeded the widespread use of ASOs as conventional medicine. One strategy that has been employed to optimize the delivery profile of ASOs, is the functionalization of ASOs with cationic amine groups, either by direct conjugation onto the sugar, nucleobase or internucleotide linkage. The introduction of these positively charged groups has improved properties like nuclease resistance, increased binding to the nucleic acid target and improved cell uptake for oligonucleotides (ONs) and ASOs. The modifications highlighted in this review are some of the most prevalent cationic amine groups which have been attached as single modifications onto ONs/ASOs. The review has been separated into three sections, nucleobase, sugar and backbone modifications, highlighting what impact the cationic amine groups have on the ONs/ASOs physiochemical and biological properties. Finally, a concluding section has been added, summarizing the important knowledge from the three chapters, and examining the future design for ASOs.

## Introduction

Antisense oligonucleotides (ASOs) are single-stranded (ss) oligomers composed of typically 10–25 nucleotides linked by negatively charged phosphorus-based linkages. ASOs have the distinctive ability to bind endogenous nucleic acid targets in a sequence-specific manner, thereby inhibiting gene expression and offering opportunities for the treatment of a broad range of diseases. As ASOs interact with their RNA (or DNA) targets through complementary Watson–Crick base-pairing, the sequence options of ASO lead compounds can be rationalized based on a knowledge of the endogenetic gene sequence to be targeted, thus further offering a potential against targets which are considered undruggable using conventional small-molecule drugs. The key features of ASOs further enable them to be transformed into personalized medicines, eventually even targeting patient-specific sequences and very rare diseases [1].

**Figure 1:**
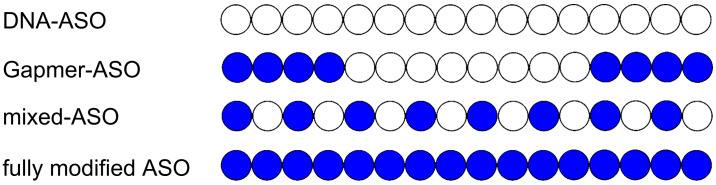
A schematic representation of 16-mer ASOs in different designs. White circles represent unmodified DNA monomers; blue circles represent nucleotide modifications. The gapmer-ASO shown is an example of a so-called 4-8-4 gapmer. The patterns of modified and unmodified nucleotides may vary and only examples are shown. Also, the phosphodiester linkages between the nucleotide monomers may be modified, and here phosphorothioate linkages are often used.

ASOs can mediate gene silencing via different mechanisms of action. ASOs that induce RNase H degradation of the endogenous RNA target generally are of the gapmer-design class (Figure 1), where a central segment of at least five DNA nucleotides termed the 'gap' is flanked by modified nucleotides that promote target binding and protection against exonucleolytic degradation [2]. Another class are the steric block ASOs that bind to the target with high affinity without inducing RNase H mediated degradation. Such ASOs are usually in part ('mixmers'), or in full, composed of nucleotides that structurally are incompatible with RNase H activity [3]. A limited number of ASOs has been approved by different agencies as medicines for the treatment of various diseases, such as Fomivirsen (1998, withdrawn), Mipomersen (2013), Eteplirsen (2016), Nusinersen (2016), Inotersen (2018), Golodirsen (2019), Volanesorsen (2019), Viltolarsen (2020), and Casimersen (2021) [3–5].

In practice, the design of ASOs that mediate efficient gene silencing without side-effects has turned out to be challenging. These side-effects have been shown to emerge due to off-target interactions [6–11], toxicities [12] or saturation of RNA-processing pathways [13]. Additionally, the delivery of ASOs to the target tissues or organs is a major hurdle that needs to be addressed before ASOs can find more widespread use [3,9–11,14]. A lack of efficient delivery of ASOs can be caused by various reasons such as degradation [15–16], insufficient endosomal escape [17], glomerular filtration [18], or binding to one or more proteins [19–20]. Notably, optimization of the therapeutic window of ASOs is closely related to improved delivery, and a variety of chemical strategies have been investigated in this context, such as ASO–lipid conjugates for improved endosomal escape [21], ASO–triantennary *N*-acetylgalactosamine (GalNAc) conjugates for improved liver targeting [22–23] and ASO–glucagon-like peptide-1 (GLP1) conjugation for improved delivery to pancreatic β-cells [24].

Previously, Menzi et al. reviewed the impact of cationic modifications and conjugations for ONs and siRNAs biophysical and biological activities until 2015 [25]. In this review, we focus on important monomeric cationic modifications for ASOs, including locked nucleic acid (LNA) monomers, and their synthesis. Such modifications have been achieved either by direct conjugation to the nucleobase, the sugar or the backbone of nucleotide monomers of such ASOs. In addition, a table design showing which modification has duplex stabilizing properties, as well as improved nuclease resistance and cell activity, has been chosen for optimal visual presentation.

This approach has spurred considerable interest since the introduction of positively charged groups results in ASOs with an overall reduced negative charge compared to the corresponding ASO without such groups. As the large number of negative charges of an ASO, i.e., *n*−1, if *n* is the number of nucleotides in an ASO with phosphodiester (PO) or phosphorothioate (PS) linkages, is assumed to contribute to the limited membrane permeability of ASOs. A reduction in the net negative charge may have beneficial delivery-related effects in addition to other possible effects such as improved resistance towards nuclease degradation or increased binding to the negatively charged RNA complements, the latter as a result of reduced electrostatic repulsion. In the following sections, a series of ASO-type oligonucleotides (ONs) which have been chemically modified with positively charged groups will be described, and their properties highlighted.

## Review

### ONs containing amine-group conjugates and nucleotide derivatives

Many parameters can affect the physicochemical properties of ASOs that have been modified with amine groups, where factors such as length and shape of the amine moiety, the attachment site of the amine group (to the sugar, backbone, or nucleobase) and the position within the ON/ASO, i.e., the 5’-terminus, 3’-terminus, or in the center. The modifications chosen for inclusion into this review involve some of the more commonly used amine groups that have been attached as a single modification either on the nucleobase, sugar, or internucleotide linkage. When structurally depicted in this paper, modifications are shown with amine functionalities in their neutral, i.e., non-protonated form.

Regarding synthetic strategies, both the use of amine group functionalized phosphoramidites, i.e., functionalized monomeric building blocks, as well as conjugation with amine groups after completion of the ON assembly, are being discussed. Amino acids and cationic modifications that replace the core structure of the nucleobase, sugar, or the internucleotide linkage have been excluded.

### Cationic amine-functionalized group substitutions at nucleobases

One strategy that has attracted a lot of interest is the attachment of cationic (poly)amine groups via the nucleobase on ASOs, thereby improving the RNA-binding affinity [26]. This strategy can be employed either on the nucleoside level, which requires many different nucleotide building blocks to be synthesized or via the so-called post-synthetic modification strategy of ONs. The latter strategy can be divided into the conjugation of amine groups onto ONs still attached to the solid support, or onto ONs in solution after cleavage from the solid support. The C-5 position of the pyrimidine ring has in general been the most used attachment point since it is not involved in hydrogen bonding and is facing the major groove upon duplex formation [27].

An illustrative approach that allowed the exploration of the 5-position of the pyrimidine ring as attachment site involved the use of 5-methoxycarbonylmethyl-2'-deoxyuridine and 5-trifluoroethoxycarbonyl-2’-deoxycytidine building blocks in the ON synthesis [28–30]. The corresponding modified ONs could be converted in a versatile manner to oligomers carrying the desired amine-functionalized groups at the 5-position on the pyrimidine nucleobase [28–30]. Similarly, the more reactive 5-cyanomethoxycarbonylmethyl-2’-deoxyuridine monomer has been used [27]. The reactivity of the above-mentioned chemical groups has enabled the attachment of various amine-functionalized groups onto the 5-position of pyrimidines via both the modified monomeric building blocks and post-synthetic ON chemistry [27–28,31–41]. The structures of some of the uridine derivatives are shown in Table 1. In general, the attachment of amine-functionalized groups on the pyrimidine C-5 position positively affects both the thermal stability and the nuclease resistance of the resulting amine-modified ONs. A tris-aminated derivative group, when conjugated to the C-5 position on 2’-deoxyuridine (Table 1B, **12**), improved antisense activity while reducing toxicity [39].

**Table 1:** Amine-functionalized groups on the nucleobase.^a^

base modifications	R^1^		*n* R^2^	Ref.	thermo- stability	nuclease resistance	activity in cell

**A** 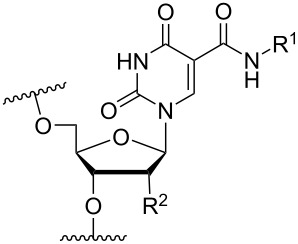	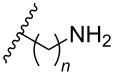	**1**	*n* = 2 R^2^ = H	[28,30]	**+**	**+**	n.d.
	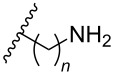	**2**	*n* = 6 R^2^ = H	[28,30–32]	**+**	**+**	n.d.
	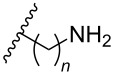	**3**	*n* = 6 R^2^ = OMe	[31,33]	**+**	**+**	n.d.
	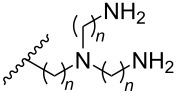	**4**	*n* = 2 or 3	[37]	**+**	**+**	n.d.

**B** 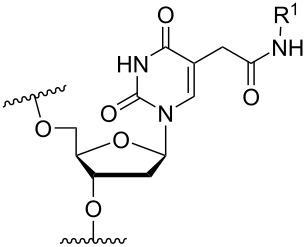	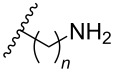	**5**	*n* = 2	[34–35]	**+**	**+**	n.d.
	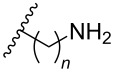	**6**	*n* = 5	[27]	n.d.	n.d.	n.d.
	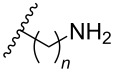	**7**	*n* = 6	[34–36]	**+**	**+**	n.d.
	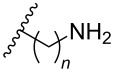	**8**	*n* = 7	[27]	n.d.	n.d.	n.d.
	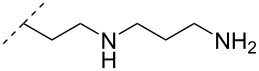	**9**		[35,38]	**+**	n.d.	n.d.
	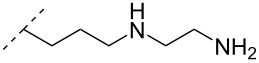	**10**		[38]	**+**	n.d.	n.d.
	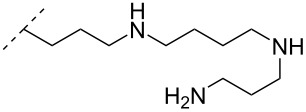	**11**		[40–41]	**+**	n.d.	n.d.
	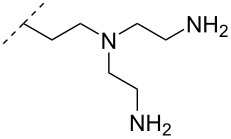	**12**		[34,39,42–43]	**+**	**+**	**X**

^a^A ‘+’ sign has been added when the modified ON/ASO showed improved thermal stability (*T*_m_) either towards ssDNA, ssRNA, or double-stranded (ds) DNA, and when the nuclease stability for the modified ON/ASO demonstrated improved stability, all compared to the effects mediated by control DNA or RNA strands. The ‘X’ sign has been added when the modified ASO demonstrated equal or better gene inhibitory activity in cells relative to the ASO control, while n.d. = not determined.

In addition, a 15-mer PS-ASO, modified with the C-5 tris-aminated 2’-deoxyuridine **12**, improved anti-HIV activity and reduced cytotoxicity relative to the unmodified PS-ASO [43]. It is important to notice that C-5 modifications, besides resulting in improved nuclease stability and providing improved target hybridization, allow for activation of RNase H. This was demonstrated by Matsuda and co-workers when they incorporated modification **2** or **3** into a 17-mer ON containing a stretch of four DNA nucleotides in the middle, flanked by the modifications in a ´mixmer´ design, which is important for designing gapmer ASOs [31].

Another well-established method for C-5 pyrimidine modification involves the Sonogashira cross-coupling reaction between an alkyne group and a 5-iodo-modified nucleobase/nucleoside followed, if desired, by reduction [44] to give a more flexible group, or the alkyne group can be retained, depending on the modification needed [45–47]. This method has been extensively used to study various modifications, and some of them can be seen in Table 2 (A and B) [44,48–54]. Interestingly, when ONs were modified with C-5 amino acid-functionalized LNA nucleotides **20**–**22**, significant increases in the melting temperatures (*T*_m_) were measured with up to 14 °C for modification **22** towards complementary RNA, relative to the unmodified DNA-ON. Furthermore, this was 5.5 °C higher than the ON modified with LNA. It is important to mention that the positioning of the modification in the 9-mer strand had a significant impact on the stability of the corresponding duplex with its RNA complement. All three modifications **20**–**22** showed better hybridization properties than LNA-thymidine; however, only modification **22** gave significant increases in *T*_m_ relative to modification **19** used as control. This finding was ascribed to both the extended π-conjugation of the alkynyl-functionalized nucleobase and stabilizing electrostatic interactions [54]. Positioning the modifications near the 3’-terminus increased the resistance toward 3’-exonuclease degradation relative to both the unmodified and the LNA-modified ONs [54].

**Table 2:** Amine-functionalized groups on the nucleobase.^a^

base modifications	R^1^		*n*/R^2^	ref.	thermo- stability	nuclease resistance	activity in cell

**A** 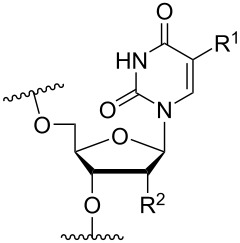	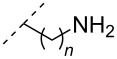	**13**	*n* = 3, R^2^ = H	[48–50]	**+**	n.d.	n.d.
	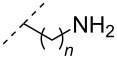	**14**	*n* = 6, R^2^ = H	[44]	**+**	n.d.	n.d.
	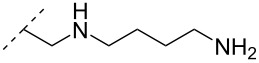	**15**	R^2^ = H	[51]	**+**	**+**	n.d.
	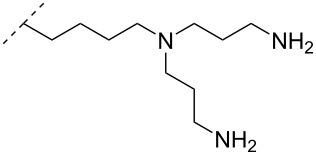	**16**	R^2^ = H	[52]	**+**	**+**	n.d.
	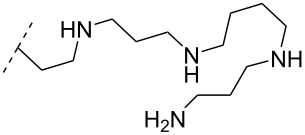	**17**	R^2^ = OH	[53]	n.d.	**+**	n.d.
	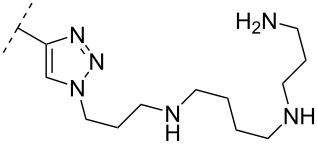	**18**	R^2^ = OMe	[62]	**+**	n.d.	n.d.

**B** 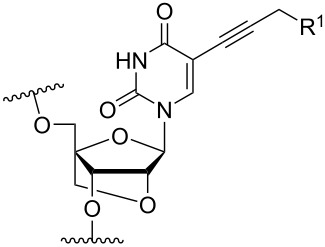	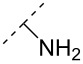	**19**		[47,55–56]	**+**	**+**	n.d.
	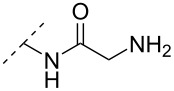	**20**		[54]	**+**	**+**	n.d.
	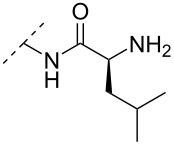	**21**		[54]	**+**	**+**	n.d.
	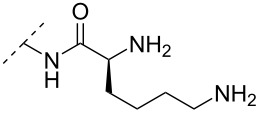	**22**		[54]	**+**	**+**	n.d.

^a^A ‘**+**’ sign has been added when the modified ON/ASO showed improved thermal stability (*T_m_*) either towards ssDNA, ssRNA or dsDNA, and when the nuclease stability for the modified ON/ASO demonstrated improved stability, all compared to the effects mediated by control DNA or RNA strands. The ‘**X**’ sign has been added when the modified ASO demonstrated equal or better gene inhibitory activity in cells relative to the ASO control, while n.d. = not determined.

Although a more simple modification regarding the chemical composition, the C5-aminopropynyl-functionalized LNA **19** has shown good duplex-stabilizing properties with up to 13 °C per modification towards RNA [47] while conferring also high triplex stability [55]. A further investigation demonstrated that C5-aminopropynyl-functionalized LNA, after being introduced into so-called bisLNAs (triplex-forming ONs (TFOs) linked to a Watson–Crick interacting ON, both targeting the same ssDNA) exhibited the ability to invade double-stranded DNA (dsDNA) targets in vitro [56].

The functionalization with aminoalkyl variants onto the nucleobase is not limited to the C-5 position on the pyrimidine base. Another important site is the C-4 position and/or a combination of both as deposed in Table 3 (A and B), with a selection of amine modifications attached. One of these is the cytosine analogue termed G-clamp (modification **23**) which increased the *T*_m_ of a DNA duplex by 18 °C when incorporated centrally into a decamer ON (Table 3A) [57]. The G-clamp modification was later observed to have antisense inhibition activity involving RNase H cleavage with a single incorporation into a PS-ON [58]. Afterwards, a guanidino-G-clamp (modification **24**) was synthesized to increase the number of hydrogen bonds that could be established between the modified nucleobase and the corresponding guanidine, which resulted in an increase in *T*_m_ of 16 °C, i.e., in the same range as obtained with the original G-clamp (Table 3A) [59].

**Table 3:** Amine-functionalized groups on the nucleobase.^a^

base modification	R^1^		*n*/R^2^	ref.	thermo- stability	nuclease resistance	activity in cell

**A** 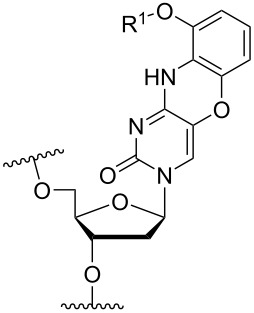	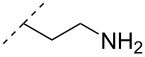	**23**		[57–58]	**+**	n.d.	**X**
	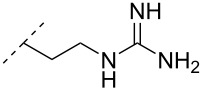	**24**		[59]	**+**	n.d.	n.d.

**B** 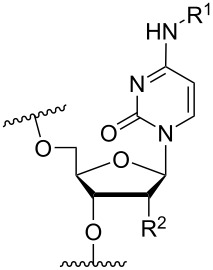	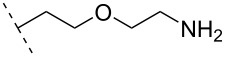	**25**	R^2^ = H	[57]	**+**	n.d.	n.d.
	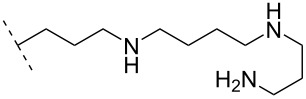	**26**	R^2^ = OMe	[61]	**+**	n.d.	n.d.
	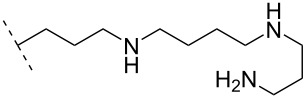	**27**	R^2^ = O-MOE	[61]	**+**	n.d.	n.d.

**C** 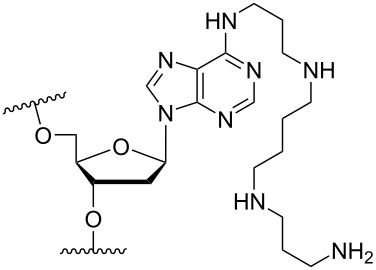		**28**		[63–65]	n.d.	n.d.	n.d.

**D** 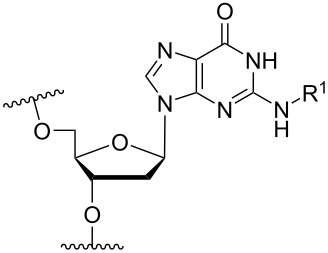	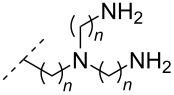	**29**	*n* = 2	[70–71]	**+**	**+**	n.d.
	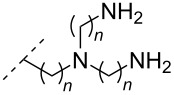	**30**	*n* = 3	[70–71]	**+**	**+**	n.d.
	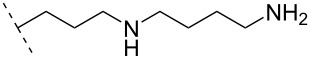	**31**		[68]	**+**	n.d.	n.d.
	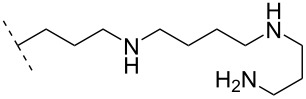	**32**		[64–69]	**+**	n.d.	n.d.

^a^A ‘**+**’ sign has been added when the modified ON/ASO showed improved thermal stability (*T_m_*) either towards ssDNA, ssRNA or dsDNA, and when the nuclease stability for the modified ON/ASO demonstrated improved stability, all compared to the effects mediated by control DNA or RNA strands. The ‘**X**’ sign has been added when the modified ASO demonstrated equal or better gene inhibitory activity in cells relative to the ASO control, while n.d. = not determined.

Generally, conversions of nucleoside phosphoramidite synthons have been explored only rarely. However, the commercially available 3’-phosphoramidite derivative of 5’-*O*-dimethoxytrityl-2’-*O*-methyluridine could be converted into an N^4^-triazole-modified 2’-OMe-cytidine phosphoramidite [60]. This concept was later used to prepare spermine-functionalized 2’-OMe and 2’-*O*-((2-methoxy)ethyl) (MOE)cytidine phosphoramidites as building blocks for incorporation into PS-ONs (Table 3B, **26**, **27**). In this study, two modifications of the N^4^-spermine-modified 2’-*O*-MOE-cytidine monomer **26** was incorporated into a 12-mer PS-ON centrally and at the 5’-end, resulting in significant increases in *T*_m_ (by more than 16 °C) towards complementary RNA [61]. In general, the study showed that the modifications had a positive effect on *T*_m_ for the formed ON/RNA duplex for all ONs substituted with cytidine monomers **26** or **27**.

These findings were also in agreement with an earlier study where the conjugation of a spermine-substituted triazole group at the C-5 position of a 2’-OMe-uridine monomer, positioning the group in the major groove of the formed duplex, had a significant stabilizing effect on RNA binding (Table 2A, **18**) [62]. Another method for introducing spermine at the N^4^ position involved the reaction of spermine and 4-*N*-*p*-toluenesulfonyl-5’-*O*-dimethoxytrityl-2’-deoxycytidine followed by ON synthesis [63].

A less investigated strategy is the anchoring of amine functionalities onto purines. Beginning with adenine, the N^6^ position has been the most explored attachment point. For example, a 2’-deoxy-N^6^-triazole-substituted adenosine monomer (9-(5’-*O*-dimethoxytrityl-2’-deoxy-β-ᴅ-erythro-pentofuranosyl)-*N*^6^-(1,2,4-triazol-4-yl)adenine) was reacted with spermine to yield 5'-*O*-dimethoxytrityl-*N*^6^-(4,9,13-triazatridecane-1-yl)-2'-deoxyadenosine (**28**) and subsequently incorporated into an ON (Table 3C) [63–65].

A different approach must be taken when conjugating amine moieties onto guanine. Interestingly, in a post-oligo synthetic modification approach, a 2-fluoro-6-*p*-nitrophenylethyl-2’-deoxyinosine-3'-phosphoramidite monomer was incorporated twice into an 11-mer ON, whereupon spermine was attached to the 2-position of the purine simultaneously with cleavage from the solid support [66]. It was found that the modified ON gave an improved duplex stability relative to the unmodified ON by 15 °C at 150 mM NaCl [66]. Later, improved syntheses of the phosphoramidite derivatives of guanine analogues have been developed [67]. Thus, many studies have been carried out for C2-spermine modified 2’-deoxyguanosine (Table 3D), and in general, when the above modification is incorporated on the C2 position, a positive and cooperative stability-enhancing effect is observed for the duplex formation between the modified ON and the targeted complementary strands [66,68–69]. Additionally, when a shorter group is introduced (spermidine, **31**, Table 3D) into a 12-mer ON, at the 5-position and at the 5’-end, a similar increase in duplex stability was observed, i.e., +22 °C for modification **31** and +21 °C for modification **32** towards complementary DNA (150 mM NaCl) [68].

It has been demonstrated that C-2 modified guanidine analogues containing nor-spermidine (**30**) and the shorter diethylenetriamine (**29**) (Table 3D) could be synthesized via the C2-fluoro modified monomer. This resulted in ONs with slightly higher *T*_m_ (approximately 3 °C for **29** and **30**) when having one incorporation relative to their unmodified versions. Additionally, the modified ONs exhibited enhanced nuclease resistance [70–71]. A recent review has recently been published with a more extensive coverage of the post-synthetic ON functionalizations [72].

### Cationic amine-functionalized group moieties attached to the sugar scaffold

The sugar moiety of ONs has been extensively studied with respect to the significance of structure and configurations of substituents, and the resulting conformations of the furanose ring, on the properties of ONs (including ASOs). The great variation at which substituents can be positioned has led to the investigation of the impact of cationic amine-functionalized groups on the biophysical properties of the resulting ONs.

One position of the sugar moiety that has been explored in detail is the 2’-position. Modifications at this site have resulted in highly therapeutically relevant monomers like 2’-OMe- and 2’-*O*-MOE-RNA [3]. Additionally, the attachment of amine-functionalized groups at the 2’-position can be readily performed and allows for the amine-functionalized group to be positioned into the minor groove of the ON duplex [73].

Aminoalkyl functionalization of the furanose sugar moiety is mainly achieved through two major pathways. The first is the synthesis of cationic nucleotide derivatives, while the second proceeds via post-synthetic chemistry on a susceptible ON. The latter strategy, in theory, allows for a more versatile approach in testing the effects of different amine-functionalized groups.

Beginning with the 2’-*O*-alkylated RNA nucleotides (Table 4) it has been shown that the introduction of an aminopropyl group via 2’-*O*-alkylation (modification **34**) leads to moderately improved hybridization properties for the modified ON against its RNA complement and improved nuclease resistance. Additionally, a 20-mer PS-ASO with nine incorporations of modification **34** near the 3’-end was introduced into A549 cells via the electroporation method to induce c-*raf* mRNA and protein knockdown. Improved activity of the modified PS-ASO relative to the unmodified PS-ASO was observed: a ten-fold higher concentration of the control PS-ASO was needed to obtain a similar knockdown effect relative to the modified PS-ASO [74]. Extending to aminohexyl (monomer **35**) resulted in a small decrease in duplex stability relative to the native ON, whereas the high nuclease resistance was maintained [75]. The attachment of a lysine onto the aminohexyl residue resulted in a lysylaminohexyl group (monomer **40**) which displayed a gradual increase in *T*_m_ upon incorporation of up to three modifications, and also improved the resistance against nuclease degradation relative to the native ON. Additionally, compared to wild‐type ONs or siRNA, ASOs carrying three modifications resulted in an equal or higher downregulation of ICAM‐1 expression [76].

**Table 4:** Amine-functionalized groups attached to the sugar scaffold.^a^

Sugar modifications	R^1^		*n*	ref.	thermo- stability	nuclease resistance	activity in cell

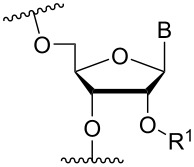	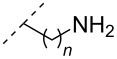	**33**	*n* = 2	[77]	n.d.	n.d.	**X**
	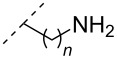	**34**	*n* = 3	[74,77–78,81]	**+**	**+**	**X**
	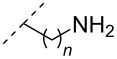	**35**	*n* = 6	[75]	n.d.	**+**	n.d.
	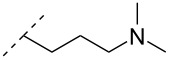	**36**		[78]	**+**	**+**	n.d.
	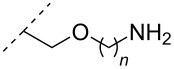	**37**	*n* = 2	[73,77]	n.d.	n.d.	**X**
	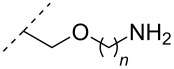	**38**	*n* = 3	[73,77]	n.d.	n.d.	**X**
	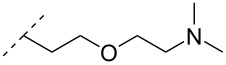	**39**		[79]	**+**	**+**	n.d.
	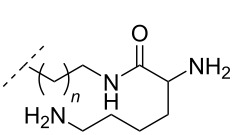	**40**	*n* = 5	[76,82–83]	**+**	**+**	**X**
	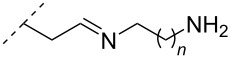	**41**	*n* = 1	[80]	**+**	n.d.	n.d.
	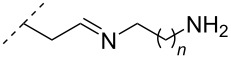	**42**	*n* = 2	[80]	**+**	n.d.	n.d.
	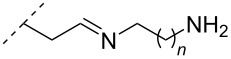	**43**	*n* = 3	[80]	**+**	n.d.	n.d.
	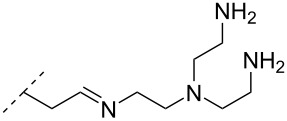	**44**		[80]	**+**	n.d.	n.d.
	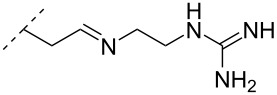	**45**		[80]	**+**	n.d.	n.d.
	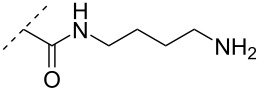	**46**		[84]	n.d.	**+**	n.d.

^a^A ‘**+**’ sign has been added when the modified ON/ASO showed improved *T_m_* either towards ssDNA, ssRNA or dsDNA and when the nuclease stability for the modified ON/ASO demonstrated improved stability compared to the DNA or RNA control. The ‘**X**’ sign has been added when the modified ASO demonstrated equal or better activity in cells relative to the ASO control, while n.d. = not determined.

A large study including 2’-aminoethyl RNA (monomer **33**) showed that two incorporations at the 3’-end of a 22-mer antisense strand of a siRNA had better eGFP gene silencing activity compared to the control siRNA with a single 2’-OMe RNA monomer near the 5’-end [77]. Conversion of the primary amine on the aminopropyl modification into a tertiary amine (monomer **36**) and insertion four times in a 16-mer ON (two in the middle and one near either end) resulted in modified ONs having a high binding affinity towards RNA relative to the affinity of the DNA-control. Afterwards, a modified 19-mer ON carrying four copies of modification **36** near the 3’-end demonstrated high nuclease stability, as also observed with modification **34** [78].

In an attempt to introduce high yielding phosphoramidite building blocks suitable for automated ON synthesis, 2′-*O*-aminoethoxymethyl and 2′-*O*-aminopropoxymethyl nucleotides were developed. This method introduced the primary amine functionality through an azide reduction [73]. The corresponding monomers **37** and **38** improved the silencing activity of a siRNA when incorporated into the passenger strand (in the eGFP assay mentioned above). However, a decrease in the silencing activity was observed when incorporated into the guide strand [77]. To design a 2’-*O-*MOE cationic analogue, the 2'-*O*-(2-(2-(*N*,*N*-dimethylamino)ethoxy)ethyl) monomer **39** has been prepared and shown to moderately enhance RNA affinity and induce high nuclease resistance, similar to that of modification **34** [79].

Optimizing the triplex stability of complexes formed between modified TFOs and their dsDNA target is an important direction of research. This has been explored utilizing the reactivity between primary amines and the aldehyde moiety of a 2’-*O*-(2-oxoethyl)uridine nucleotide, incorporated centrally in an 11-mer TFO, to form a Schiff base (monomers **41**–**45**) [80]. All aminoalkylated moieties improved the triplex stability. Notably, a significant improvement in *T*_m_ was observed when the functionalizing groups were changed from ethylenediamine to either trimethylenediamine (monomer **42**) or putrescine (monomer **43**), demonstrating that the length between the cationic amino group and the sugar scaffold is important for the thermal stability effects. The best stabilization was obtained for the 2-(aminoethyl)guanidine monomer **45** and tris(2-aminoethyl)amine (monomer **44**) variants [80].

Another chemical group utilized for the 2’-modification is 2’-*O*-carbamoyl [85–87]. However, it has proven difficult to stabilize the duplex formed between the modified ON and targeted DNA/RNA with cationic aminoalkylated groups [85], which is thought to be due to the close contact between the carbonyl of the 2’-*O*-carbamoyl substituent and the O-2 on the nucleobase [88]. To circumvent this issue, the 2'-*O*-(*N*-(4-aminobutylcarbamoyl))uridine monomer **46** has been synthesized [84]. When incorporated centrally in a 2’-OMe-RNA ON, a significant stabilization relative to that of 2′-*O*-carbamoyluridine was observed against the RNA target. However, relative to the control ON carrying pure 2’-OMe RNA modified ONs, monomer **46** had an affinity-lowering effect [84]. A nuclease stability assay showed a clear improvement relative to the control and the simple 2′-*O*-carbamoyluridine modification [84].

A different approach to introduce aminoalkyl groups at the 2’-position was achieved via a benzyl protected 2’-succinylamido-2’-deoxyuridine building block attached either to a solid support or incorporated using conventional phosphoramidite chemistry. Previously such a method had been used to attaching different moieties onto an ON still bound to a solid support [89–90]. Putrescine (**47**), spermidine (**48**), spermine (**49**) and a synthetic pentaamine (**50**) were attached to the 2’-position (Table 5).

**Table 5:** Amine-functionalized groups attached to the sugar scaffold.^a^

sugar modifications		R^1^		ref.	thermo- stability	nuclease resistance	acitivty in cell

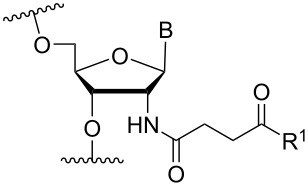		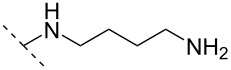	**47**	[91]	n.d.	n.d.	**X**
	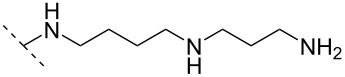		**48**	[91]	n.d.	n.d.	**X**
	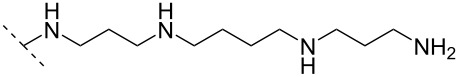		**49**	[91]	**+**	n.d.	**X**
	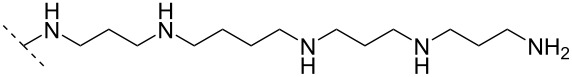		**50**	[91]	**+**	n.d.	**X**

^a^A ‘**+**’ sign has been added when the modified ON/ASO showed improved *T_m_* either towards ssDNA, ssRNA or dsDNA and when the nuclease stability for the modified ON/ASO demonstrated improved stability compared to the DNA or RNA control. The ‘**X**’ sign has been added when the modified ASO demonstrated equal or better activity in cells relative to the ASO control, while n.d. = not determined.

Interestingly the resulting nucleotides were found to adopt a conformation which was, with respect to duplex stability, tolerated at terminal positions but not at internal positions [91]. The modified PS-ASOs were transfected into human-607B melanoma cells, and after 48 h, the B-cell lymphoma 2 (bcl-2) protein levels were examined. All PS-ASOs gave improved downregulation relative to the control (scrambled sequence), but only PS-ASOs carrying the nucleotides modified with spermine (i.e., monomer **49**) or pentaamine (i.e., monomer **50**) induced improved downregulation of gene expression relative to the downregulation of the reference ASO (Oblimersen) [91].

In an entirely different approach, the 2’-amino group of amino-LNA-thymine (amino-LNA-T) has been explored as an attachment point for various cationic groups. As one example, amino acids such as glycine, lysine, and proline in different combinations have been attached to the 2’-amino group with substantial success regarding duplex stability [92]. The 2’-amino-LNA scaffold has further been modified with amine-functionalized groups at the nucleoside level creating different nucleotide building blocks for ON synthesis, or at the ON level via post-ON synthesis conjugation chemistry. The first method was used to attach 1-piperazinepropionic acid through an amide coupling onto 2’-amino-LNA. This monomer (**51**) induced high binding affinity towards complementary targets upon incorporation into a 9-mer ON. In DNA, an increase of 7.0 °C and 17.5 °C for one and three incorporations, respectively, was observed and in RNA, an increase of 9.0 °C and 24.5 °C for one and three incorporations, respectively, was observed relative to the DNA 9-mer control strand. Additionally, a high nuclease resistance compared to the DNA control was observed [93]. In a follow-up study, after being introduced into bisLNAs, the piperazino-modified 2'-amino-LNA-T nucleotide was compatible with invasion into dsDNA targets in vitro [56]. In further studies utilizing the same amide coupling, nor-spermidine with different group lengths (**52** and **53**), a glycol-amine-functionalized group (**54**), and a bis-C6-amine-functionalized group (**55**) were attached to 2’-amino-LNA-T. All these modifications demonstrated high duplex stabilizing capabilities combined with high nuclease resistance [94–95]. Additionally, the nor-spermidine and amino-glycol modified 2’-amino-LNA-T when incorporated into TFOs all induced excellent triplex stability at pH 7.0 [94] (Table 6).

**Table 6:** Amine-functionalized groups attached to the sugar scaffold.^a^

sugar modifications	R^1^		*n*/R^2^	ref.	thermostability	nuclease resistance	activity in cell

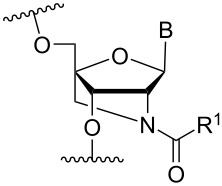	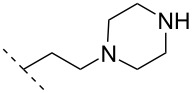	**51**		[93]	**+**	**+**	n.d.
	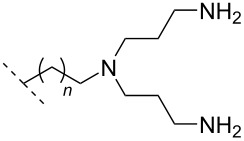	**52**	*n* = 1	[94]	**+**	**+**	n.d.
	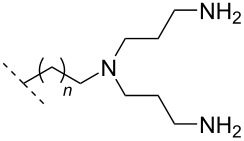	**53**	*n* = 2	[94]	**+**	**+**	n.d.
	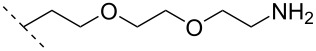	**54**		[94]	**+**	**+**	n.d.
	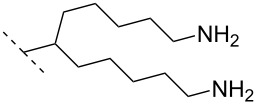	**55**		[95]	**+**	**+**	n.d.
	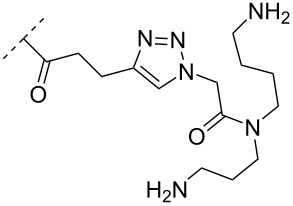	**56**		[95]	**+**	**+**	n.d.
	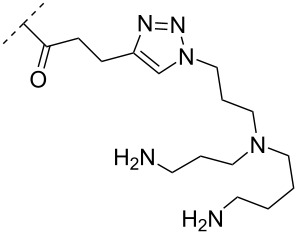	**57**		[95]	**+**	**+**	n.d.
	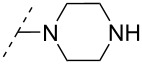	**58**		[96]	**+**	n.d.	n.d.
	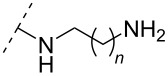	**59**	*n* = 1	[96]	**+**	n.d.	n.d.
	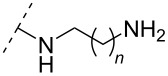	**60**	*n* = 2	[96]	**+**	n.d.	n.d.
	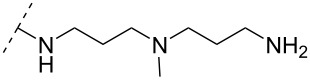	**61**		[96]	**+**	n.d.	n.d.
	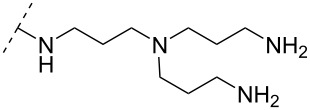	**62**		[96]	**+**	n.d.	n.d.

^a^A ‘**+**’ sign has been added when the modified ON/ASO showed improved *T_m_* either towards ssDNA, ssRNA or dsDNA and when the nuclease stability for the modified ON/ASO demonstrated improved stability compared to the DNA or RNA control. The ‘**X**’ sign has been added when the modified ASO demonstrated equal or better activity in cells relative to the ASO control, while n.d. = not determined.

To circumvent the laborious work related to the monomers described above, post-ON conjugation via click-chemistry was utilized to attach two different spermidine analogues, carrying either two (**56**) or three (**57**) positive charges, onto the 2’-amino-LNA scaffolds. This was demonstrated to be a successful design as both monomers showed very high duplex stabilizing properties towards RNA (*T*_m_ +10.0 °C) and DNA (*T*_m_ +8.5 °C). Additionally, nuclease resistance was shown to be high [95] which was in agreement with other 2’-aminoalkylated-LNA monomers [94]. The triplex stability of the monomers was determined, and the spermidine variant carrying three cationic charges (**57**) had the highest triplex stabilizing effect [95]. Interestingly, at biologically relevant pH (7.0), two incorporations of the nor-spermidine variant (**53**) [94], and the triazole-linker variant carrying three cationic charges (**57**) [95] stabilized the formed triplex by 28.0 °C and 30.5 °C, respectively [94–95]. These cationic 2’-amine-functionalized LNA modifications (**51**–**57**) all gave high binding affinity towards RNA with excellent nuclease resistance, making them ideal ASO modifications as only a limited number of modifications is needed for a substantial effect.

Recently, a versatile method of post-ON synthesis conjugation, different from the click chemistry method, has been applied to 2’-amino-LNA. A class of 2’-urea-LNA analogues (**58–62**) has been prepared by reacting various amine-functionalized groups with a 2'-*N*-pentafluorophenoxycarbonyl-2'-amino-LNA monomer already incorporated into an ON. All modifications improved hybridization towards both DNA and RNA complements when compared to natural DNA nucleotides [96].

The attachment of an aminoalkyl-group to the 4’-position (Table 7A) offers an advantage since this site is in close proximity to the backbone, which potentially allows the basic amino group via a relative short linking group to engage electrostatically with the acidic phosphodiester moiety [97]. This was initially explored for ONs containing 4′-*C*-(aminomethyl)thymidine (monomer **63**) [98–99]. Later, the amine-functionalized group was expanded into the monomer 4′-*C*-(2-((*N-*(2-aminoethyl)carbamoyl)oxy)ethyl)thymidine (not shown) that was shown to display improved resistance against endo- and exonuclease cleavage relative to the DNA control ON [100]. This was followed by 4′-*C*-amidoethyl- (**64**) and 4′-*C*-amidopropylthymidine (**65**) derivatives which continued the trend of good nuclease stability. In general, all the 4’-substituted nucleotides (**63–66**) mentioned here stabilized duplexes formed with DNA complements with modification **64** giving the best stabilization (up to 5.7 °C for four incorporations in an 18-mer ON relative to the control), whereas all gave similar and/or decreased thermal stability against RNA relative to the natural control ON [97]. It is noteworthy to mention that all monomers allowed the design of ASOs that were substrates for RNase H [97].

**Table 7:** Amine-functionalized groups attached to the sugar scaffold.^a^

sugar modifications	R^1^		*n*/R^2^	ref.	thermo- stability	nuclease stability	activity in cell

**A** 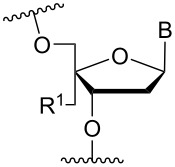	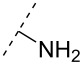	**63**		[97–99]	**+**	**+**	n.d.
	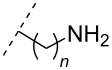	**64**	*n* = 1	[97]	**+**	**+**	n.d.
	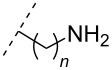	**65**	*n* = 2	[97]	**+**	**+**	n.d.
	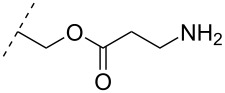	**66**		[97]	**+**	**+**	n.d.

**B** 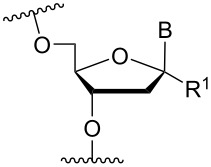	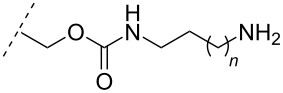	**67**	*n* = 2	[101]	**+**	**+**	n.d.
	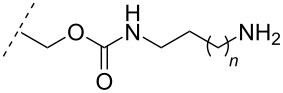	**68**	*n* = 4	[101]	n.d.	n.d.	n.d.

^a^A ‘**+**’ sign has been added when the modified ON/ASO showed improved *T_m_* either towards ssDNA, ssRNA or dsDNA and when the nuclease stability for the modified ON/ASO demonstrated improved stability compared to the DNA or RNA control. The ‘**X**’ sign has been added when the modified ASO demonstrated equal or better activity in cells relative to the ASO control, while n.d. = not determined.

The 1’-position on the furanose ring has been studied to a lesser degree regarding the functionalization by amine-containing moieties. This site allows for the substitution to be positioned towards the minor groove. To develop a new way of attaching various functional groups onto the ONs without disturbing duplex formation, Matsuda and co-workers developed some 2’-deoxyuridine analogues carrying aminoalkyl groups at the 1'-position (Table 7B). These were intended to be used as an attachment point, and the 1’-aminobutane variant of the 2’-deoxyuridine analogue (monomer **67**) was also tested for the duplex forming capabilities. Here it was observed that this modification positioned at the 5’-end in a poly-T ON stabilized the duplex (+3.0 °C relative to the control ON) whereas a central insertion of modification **67** or **68** had a neutral or slightly negative effect on the *T*_m_ value relative to the control ON [101].

### Cationic amine-functionalized group functionalities as internucleoside linkage

Although previous research has highlighted the relevance of the phosphodiester-linked backbone in the overall function of nucleic acids [102–103], many researchers have still sought to change the properties of ONs, i.e., enzymatic stability, hybridization, biodistribution, and cell-uptake, via the introduction of non-natural internucleoside linkages. The most well-known modification is the phosphorothioate-linked backbone, which is known to enhance not only nuclease resistance but also protein interactions compared to the phosphodiester backbone [104]. In an effort to reduce the overall negative charge of the backbone, also a large number of different artificially linked backbone ONs has been synthesized, and a selected number of these can be seen in Table 8 and Table 9. The latter approach of modifying the internucleotide linkage is unique as it may reduce in part or in full the negative charge of ONs, including ASOs.

One strategy that has been employed is the aminoalkyl phosphoramidate linkage (Table 8). Pioneering work was done by Letsinger and co-workers in 1986, who reported the synthesis of a 2’-deoxyadenosyl dinucleotide linked via an aminoethyl phosphoramidate linkage that was positively charged under neutral to acidic conditions [105]. A subsequent work based on these findings resulted in the synthesis of short cationic DNA ONs linked via *N*-alkylated phosphoramidate linkages (Table 8) [106]. In contrast to the dinucleotide which was synthesized by solution phase chemistry [105], the modified ONs were synthesized on solid-support employing H-phosphonate chemistry, followed by the oxidative coupling with the appropriate diamines to give the desired *N*-ethyl-2-morpholino- (monomer **71**) and *N*-methyl-2-(dimethylamino)ethyl (monomer **72**) phosphoramidate linkages [106]. How these *N*-alkylated phosphoramidate-linked ONs interacted with the complementary DNA was highly dependent on the ionic strength and the pH of the relevant medium. An inverse effect between hybridization stability and salt concentration was observed for cationic ONs when compared to their anionic counterparts. The study demonstrated a decrease in hybridization for the phosphoramidate-modified ONs towards complementary DNA when a high salt concentration (1.0 M NaCl) was used, caused by electrostatic shielding mediated by the salt ions [106]. Additionally, this class of ONs showed high resistance towards nuclease-catalyzed degradation [106].

**Table 8:** Amine-functionalized groups as internucleoside linkage.^a^

backbone modifications	R^1^		α/β	*n*/R^2^	ref.	thermo- stability	nuclease resistance	activity in cell

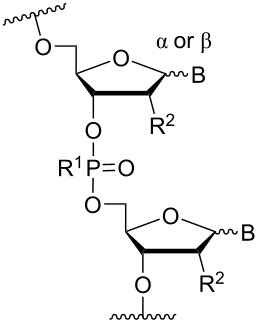	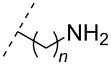	**69**	β	*n* = 1 R^2^ = H	[113]	**+**	**+**	n.d.
	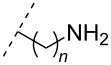	**70**	β	*n* = 2 R^2^ = H	[114]	**+**	**+**	n.d.
	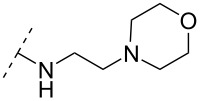	**71**	α/β	R^2^ = H	[106]	**+**	**+**	n.d.
	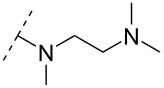	**72**	α/β	R^2^ = H	[106]	**+**	**+**	n.d.
	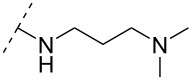	**73**	α	R^2^ = H	[107,109–110,112]	**+**	n.d.	**X**
	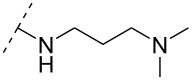	**74**	α/β	R^2^ = OMe	[107]	**+**	n.d.	n.d.
	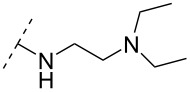	**75**	α	R^2^ = H	[108,111,116]	**+**	**+**	**X**
	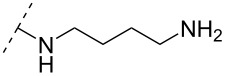	**76**	α	R^2^ = H	[112]	**+**	n.d.	n.d.
	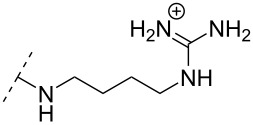	**77**	α	R^2^ = H	[112]	**+**	n.d.	n.d.
	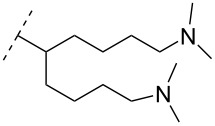	**78**	β	R^2^ = H	[115]	n.d.	n.d.	n.d.
	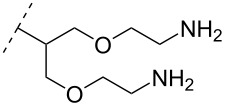	**79**	β	R^2^ = H	[115]	**+**	n.d.	n.d.
	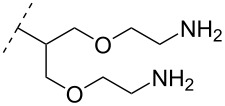	**80**	β	R^2^ = OMe	[115]	n.d.	n.d.	n.d.
	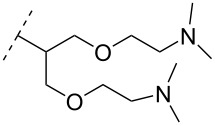	**81**	β	R^2^ = OMe	[115]	**+**	**+**	**X**

^a^A ‘**+**’ sign has been added when the modified ON/ASO showed improved thermal stability (*T_m_*) either towards ssDNA, ssRNA or dsDNA, and when the nuclease stability for the modified ON/ASO demonstrated improved stability, all compared to the effects mediated by control DNA or RNA strands. The ‘**X**’ sign has been added when the modified ASO demonstrated equal or better gene inhibitory activity in cells relative to the ASO control, while n.d. = no determined.

Subsequently, two variants of this phosphoramidate linker strategy were synthesized, one being the *N,N-*(dimethylaminopropyl)phosphoramidate linkage (monomers **73** and **74**) (DMAP) [107], and the other the *N*,*N*-diethyl-ethylenediamine phosphoramidate linkage (**75**) (DEED) [108]. Stereo-uniform ONs (either *R*_p_ or *S*_p_) containing the DMAP modification were synthesized via dinucleotide derivatives obtained by a phosphitylation reaction followed by oxidative amidate coupling to create an epimeric mixture of the dinucleotide phosphoramidate-linked derivatives with subsequent separation of the two stereoisomers. These were then incorporated into the desired ONs after O3’-desilylation and phosphitylation of the dimers [107]. The authors found that for each of the sequences investigated in the study, one phosphoramidate stereoisomer induced improved hybridization towards targeted DNA, while the other stereoisomer induced lower affinity, all relative to their corresponding phosphodiester control ON [107].

Vasseur, Debart and co-workers introduced the DMAP modification into α-configured ONs [109–110]. These zwitterionic or cationic α-ONs hybridized with high affinity to their complementary DNA and RNA targets, while a significant impairment in hybridization was found when mismatches were introduced [109–110]. This was especially seen in case of a 12-mer α-ON containing 11 modifications of the DMAP linkage, giving the ON an overall net charge of +11, resulting in a *T*_m_ increase of 27.0 °C for the ON/DNA duplex and 10.4 °C for the ON/RNA duplex [110]. A fully DMAP-modified 18-mer α-ASO was incubated with HePG2 hepatoma cells, inhibiting firefly luciferase activity in a dose-depending manner in a whole cell assay, while the scrambled control showed no effect [110]. Interestingly, this effect was observed without any transfection agents. However, the isosequential 2'-OMe ASO and the methoxyethylphosphoramidate (PNHME) ASO (with a neutral backbone) showed no activity when they were incubated without a transfection agent [110]. The β-configured ON variants resulted in a decrease in *T*_m_ towards their complementary RNA and DNA targets, which was ascribed to increased steric hindrance [109], although an improved triplex stability for the 2’-OMe RNA phosphoramidate variant was observed (**75**).

The cationic phosphoramidate variant termed DEED was originally tested for its triplex-forming capabilities. The authors found that ONs containing the DEED modification were more capable at forming triplexes under conditions that approximated the magnesium, pH, and potassium levels found in vivo [108]. A later study conducted by Weeks and co-workers reported that a TFO modified with the DEED modification could efficiently inhibit the expression of plasmid DNA injected into *Xenopus oocytes* [111]. The study demonstrated that a sufficiently long mismatch-free DNA target needed to be present for the modified TFO to work effectively, thus demonstrating the significance of sequence-specific binding. It was however important that the TFO and plasmid were mixed prior to injection, to get near-complete inhibition of gene expression. Only partial inhibition could be observed, if TFOs were injected before the plasmid, and no inhibition could be observed when the plasmid was injected first. This indicated that a competition between the cationic TFOs and the histones for DNA binding had a large impact [111].

The library of phosphoramidate variants was expanded when the aminobutyl phosphoramidate and the guanidinobutyl phosphoramidate were synthesized [112]. A facile post-synthetic method was successfully employed to convert amine functionalities attached to ONs into guanidinium tethers. Both, the aminobutyl (**76**) and guanidinobutyl (**77**) modifications were introduced into α-ONs which elicited significant stabilization of the formed ON/DNA and ON/RNA duplexes. Interestingly, towards RNA complements, these modifications resulted in more pronounced increases in *T*_m_, relative to the DMAP modification mentioned above, with the guanidino phosphoramidate modification providing a 14.0 °C increase in *T*_m_ relative to the DMAP modification for fully modified ONs [112]. Furthermore, a noticeable increase in *T*_m_ was observed for all fully modified α-TFOs irrespectively of modification (DMAP, aminobutyl or guanidinobutyl phosphoramidate) relative to the unmodified β-TFO control [112]. A cell uptake assay was conducted between two 12-mer poly-T ASOs, one being an α-ASO with a fully modified guanidinobutyl phosphoramidate backbone and the other a PS-β-ASO control, both fluorescein-labelled at the 5’-end. The study found that the guanidinium modification increased the cellular uptake. However, ASOs carrying the novel guanidinium modification were mainly localized in the cytoplasm, indicating that the ASOs are taken up by endocytosis but are retained in part in the endocytic vesicles [112].

Utilizing the phosphorus atom as an attachment point for cationic aminoalkyl groups has been employed with slight variations. Fathi, Cook and co-workers successfully introduced aminomethyl phosphonate [113] (**69**) and aminoethyl phosphonate [114] (**70**) linkages (Table 8). The introduction of the stereo-pure aminomethyl phosphonate linkage was achieved by preparing the appropriate stereo-pure (*R*_p_ or *S*_p_) thymidine dinucleotide linked through the 3’-5’ oxygen atoms modified with the phthalimidomethyl phosphonate linkage [113]. Later, a halogenated phthalimide protection was employed for the synthesis of the amidoethyl phosphonate variant [114]. The desired dinucleotides were phosphitylated and incorporated into 13-mer poly-T ONs [113–114]. For both modifications, the ONs modified with the *R*_p_-isomer formed more stable duplexes with DNA and RNA complements relative to the control ON. The ONs carrying the *S*_p_-isomer had a destabilizing effect. When tested for their nuclease resistance, an increase in stability was observed relative to their unmodified ON [113–114]. Interestingly, a difference in hydrolysis rate was noticed: the aminomethyl (**69**) modification was readily hydrolysed at pH 7 whereas the aminoethyl (**70**) modification was completely stable under the same conditions [114]. A preliminary cell uptake assay was conducted for a net-neutral ASO carrying the aminoethyl phosphonate linkage. This showed that under appropriate conditions (1 µM and at 37 °C) the net-neutral ASO had improved uptake relative to the anionic PO-ASO, demonstrating a concentration-dependent uptake [114].

A new class of internucleotide linkages has recently been introduced, termed branched charge-neutralizing sleeves (BCNSs). These cationic internucleotide linkages were synthesized through conventional phosphoramidate chemistry with a slight variation. In contrast to the standard method, bis(diisopropylamino)chlorophosphine was first reacted with either of the three diaminoalcohol groups, before subsequent phosphitylation with the DMT-protected nucleosides [115]. After incorporation into ONs, conversion to monomers **78–81** was accomplished as shown in Table 8. Although monomer **78** showed good yield in both phosphoramidite synthesis and coupling efficiency on the synthesizer, significant loss of the hydrocarbon-linked group was observed during the alkaline deprotection conditions [115]. After insertion into a 19-mer DNA-ON, modification **79** did not show any significant increase in *T*_m_. This trend was changed when the modification **81** was inserted into a 21-mer 2’-OMe RNA-ON and hybridized to a complementary 2’-OMe RNA complement. Relative to the unmodified 2’-OMe RNA sequence, the modified ON carrying six insertions of monomer **81** gave an increase in *T*_m_ of 11 °C. The serum stability of modification **81** was evaluated in PBS/bovine serum at 37 °C for 8 h. Here, a significant improvement over the unmodified 2’-OMe RNA was observed for modification **81** [115]. The two BCNSs used for monomers **79–81** (1,3-bis(2-(amino)ethoxy)-2-propyl and bis(2-(dimethylamino)ethoxy)-2-propyl) were tested for their cell uptake properties. The BCNS carrying the dimethylamino groups had a higher effect on the cellular uptake for 5’-FAM-labelled ASOs relative to the oligomers carrying BCNS being primary amines. However, both modifications demonstrated an improved cellular uptake relative to the unmodified 5’-end FAM-labelled 2’-OMe ASO [115].

Another strategy to introduce cationic aminoalkylated moieties onto phosphorus atoms of the ON backbone involves aminoalkylated phosphorothioate linkages. In general, modifications of the backbone have been used in the context of the phosphodiester linkage, while only a few examples can be found for the PS linkage [117–121]. Rahman, Obika and co-workers investigated the properties of ONs modified with the aminoalkyl–PS linkage [117], based on the post-synthetic alkylation protocol developed earlier by Chen and Gothelf reacting PS-ONs with 2-bromoethylammonium bromide in HEPES (4-(2-hydroxyethyl)-1-piperazine-ethanesulfonic acid) buffer (dimethylformamide/H_2_O 1:9) at 45 °C [118]. Initially, a 12-mer sequence containing the nucleobases guanine, thymine, and cytosine was tested by incorporating the earlier reported aminoethyl–PS linkage [118] (modification **82**). However, cleavage products formed by guanine alkylation prompted a switch to a 12-mer sequence containing only cytosine and thymine [117].

Conjugation of the desired aminoalkyl moieties with the stereo-pure PS-ON (*R*_p_ or *S*_p_) gave the aminoalkyl–PS linkages shown in Table 9A, in yields between 24–55%. Extensive work has been carried out for the synthesis of stereochemically pure PS-ONs and one of these methods employs the proline-derived bicyclic oxazaphospholidine monomer [122] which was later used in the scalable synthetic process of therapeutic stereopure PS-ASOs [123]. When the modified ONs were hybridized with their complementary DNA strands it was clear that the *R*_p_ stereoisomer of the aminoalkylated PS linkage improved the stability of the formed duplexes, while the *S*_p_ stereoisomer destabilized the formed duplexes, all relative to their PS-ON controls. Against the complementary RNA target, the *R*_p_ stereoisomer either had a similar or slightly lower *T*_m_ than the control ON, while the *S*_p_ stereoisomeric linkages destabilized the formed aminoalkylated PS-ON/RNA duplex significantly. When tested for their triplex-forming properties, a similar pattern was observed. The *R*_p_ stereoisomeric linkages gave an improved stabilization of up to +6 °C, with the non-cyclic diamine (monomers **87** and **88**) stabilizing duplex formation the most, while all *S*_p_ stereoisomeric linkages gave lower *T*_m_ than their control [117]. The nuclease stability of the aminoethyl (**82**) and aminohexyl (**86**) PS linkages was tested relative to the PS-ON control. Interestingly, both modifications showed an improved resistance relative to the control ON; however, the *S*_p_-aminoethyl (**82**) PS-linkage was slightly better than the *S*_p_-aminohexyl (**86**) linkage while the opposite was true for the *R*_p_ stereoisomeric linkages [117]. It is noteworthy to mention that the 2-(3-aminopropyl)aminoethyl (**88**) PS linkage was too heat-labile for nuclease tests [117].

**Table 9:** Amine-functionalized groups as internucleoside linkage.^a^

backbone modifications	R^1^		*n*/R^2^	ref.	thermo- stability	nuclease resistance	activity in cell

**A** 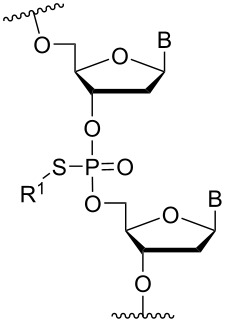	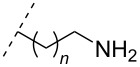	**82**	*n* = 1	[117–118]	**+**	**+**	n.d.
	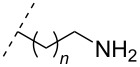	**83**	*n* = 2	[117]	**+**	n.d.	n.d.
	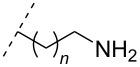	**84**	*n* = 3	[117]	**+**	n.d.	n.d.
	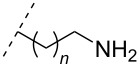	**85**	*n* = 4	[117]	**+**	n.d.	n.d.
	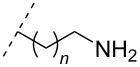	**86**	*n* = 5	[117]	**+**	**+**	n.d.
	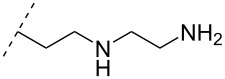	**87**		[117]	**+**	n.d.	n.d.
	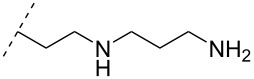	**88**		[117]	**+**	n.d.	n.d.
	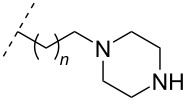	**89**	*n* = 1	[117]	**+**	n.d.	n.d.
	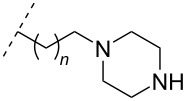	**90**	*n* = 2	[117]	**+**	n.d.	n.d.

**B** 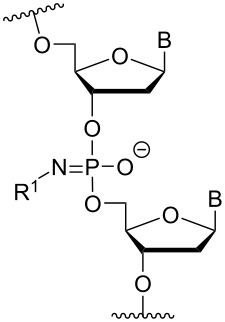	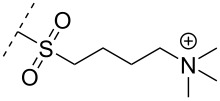	**91**		[124]	**+**	**+**	n.d.

^a^A ‘**+**’ sign has been added when the modified ON/ASO showed improved thermal stability (*T_m_*) either towards ssDNA, ssRNA or dsDNA, and when the nuclease stability for the modified ON/ASO demonstrated improved stability, all compared to the effects mediated by control DNA or RNA strands. The ‘**X**’ sign has been added when the modified ASO demonstrated equal or better gene inhibitory activity in cells relative to the ASO control, while n.d. = no determined.

Filichev and co-workers have recently synthesized a variant of the phosphoramidate internucleotide linkage (Table 9B) and have obtained some preliminary results. Modification **91** was introduced via Staudinger reaction during solid-phase DNA synthesis, circumventing some of the laborious work involved in the synthesis of some internucleotide linkages. The authors demonstrated that this new modification could enhance the stability of the ON/RNA duplex, although no significant change was observed for the ON /DNA duplex (100 mM NaCl). Additionally, the dependency of the position of the modification had a large effect, since changing the position, for a single modification from the 5’-end to the middle and then to the 3’-end, resulted in different *T*_m_ increases of 7 °C, 1 °C, and 12 °C, respectively, for the ON/RNA duplex, relative to the DNA control [124]. At pH 5.0, the internucleotide linkage could stabilize a triplex between 10–11 °C when the modification was positioned in the middle, near the 3’-end or as a double-modified TFO near the 5’- and 3’-ends, although this high stability diminished when three or four modifications were inserted [124]. At pH 6.0 no significant stabilization was observed; however, a good nuclease stability was observed for the ON carrying four insertions of modification **91** [124].

The concept of introducing cationic backbone linkages extends beyond conjugating cationic aminoalkyl groups to the phosphorus atom which, however, is beyond the scope of this summary. For a more in-depth account of cationic backbone modifications, we direct the reader to a recent review by Meng and Ducho [125].

## Conclusion

Cationic amine- and polyamine-conjugates/derivatives have the potential to improve the properties of ASOs for defined applications. Many different aspects need to be considered when optimizing an ASO for a defined application. This includes the position of a modification as well as the chemical composition of the cationic group, but also what scaffold the cationic group is to be attached to, i.e., is it a 2’-amino-LNA nucleotide or the common DNA/RNA nucleotides. This difference is reflected when evaluating groups attached to the base and sugar moieties. When the cationic group is connected via the nucleobase, thus allowing the group to be positioned in the major groove of the duplex, an increased target binding affinity is usually seen relative to modifications on the furanose ring (minor groove). However, when the 2’-amino-LNA scaffolds is used, a very high duplex stability is seen irrespectively of the target (DNA or RNA), as a synergistic effect between the locked sugar conformation and the cationic moiety emerges. Additionally, sugar modifications tend to bring higher nuclease resistance compared with nucleobase modifications. However, a drawback is the lack of RNase H activation for most of the sugar-modified derivatives. This is contrary to the nucleobase-modified variants that are in general well tolerated RNase H substrates. Another important aspect is the overall net-charge and the charge density of the ON. As the sugar and nucleobase substitutions compensate for the anionic charge carried by the PO and PS-backbone, a more densely charged ON is created which can result in synthetic difficulties. This can be circumvented by using internucleotide linkage modifications which generate net neutral or net positive ASOs, which usually have a high resistance towards nuclease degradation. Additionally, the internucleotide modifications result in ASOs that can stabilize the ASO/RNA duplex to a relatively high degree, although the *R*_p_ stereoisomer is generally the preferred isomer for improved ASO/RNA stabilization. These considerations relate to the overall design of the ASOs, since the gapmer design allows for sugar-modified nucleotides to be used on the flanks, while for a mixmer- or a fully modified ASO a more diverse composition might be used.

**Figure 2:**
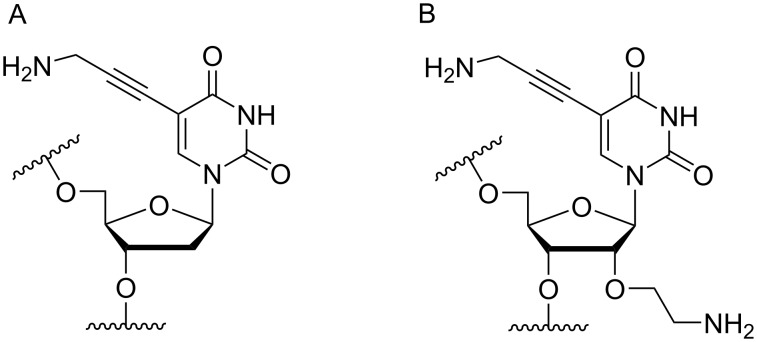
Structures of 5-(1-propargylamino)-2’-deoxyuridine (A) and 2’-aminoethoxy-5-propargylaminouridine (B).

Another consideration is the use of more densely modified nucleotides, i.e., carrying both modified base and sugar moieties. Although not as extensively explored, this strategy was employed by Fox and co-workers who elaborated on a 2’-deoxyuridine variant carrying a 1-propargylamino group (Figure 2A) that had already demonstrated enhanced stability for the T·AT triplet [126–127]. This monomer was further modified to a bis-modified uridine analogue, i.e., a nucleotide containing both the 1-propargylamino group on the 5-position and an aminoethoxy moiety at the 2’-position (Figure 2B), thus creating a monomer that could further stabilize the triplex [128]. In theory, this strategy allows for fewer modifications to be used in designing ASOs with high RNA target affinities.
